# Miscibility of rock and ice in the interiors of water worlds

**DOI:** 10.1038/s41598-022-16816-w

**Published:** 2022-07-29

**Authors:** Tanja Kovačević, Felipe González-Cataldo, Sarah T. Stewart, Burkhard Militzer

**Affiliations:** 1 Department of Earth and Planetary Science, University of California, Berkeley, CA 94720 USA; 2 Department of Earth and Planetary Sciences, University of California, Davis, CA 95616 USA; 3 Department of Astronomy, University of California, Berkeley, CA 94720 USA

**Keywords:** Exoplanets, Structure of solids and liquids, Thermodynamics

## Abstract

Super-Earths and sub-Neptunes are the most common planet types in our galaxy. A subset of these planets is predicted to be water worlds, bodies that are rich in water and poor in hydrogen gas. The interior structures of water worlds have been assumed to consist of water surrounding a rocky mantle and iron core. In small planets, water and rock form distinct layers with limited incorporation of water into silicate phases, but these materials may interact differently during the growth and evolution of water worlds due to greater interior pressures and temperatures. Here, we use density functional molecular dynamics (DFT-MD) simulations to study the miscibility and interactions of enstatite (MgSiO_3_), a major end-member silicate phase, and water (H_2_O) at extreme conditions in water world interiors. We explore pressures ranging from 30 to 120 GPa and temperatures from 500 to 8000 K. Our results demonstrate that enstatite and water are miscible in all proportions if the temperature exceeds the melting point of MgSiO_3_. Furthermore, we performed smoothed particle hydrodynamics simulations to demonstrate that the conditions necessary for rock-water miscibility are reached during giant impacts between water-rich bodies of 0.7–4.7 Earth masses. Our simulations lead to water worlds that include a mixed layer of rock and water.

## Introduction

The discovery of thousands of exoplanets by the *Kepler*, *CoRoT*, and *TESS* space telescope missions have revealed a plethora of planetary types within our galaxy^1^. From measurements of planetary radii, planets ranging in size between Earth and Neptune have emerged as the most common planet type in our galaxy^2^. These planets, known as super-Earths and sub-Neptunes, have no analog in our solar system, and little is known about their interior composition, which is crucial for understanding their formation and evolution. In order to explain the observed masses and radii, hydrostatic equilibrium models have been developed to infer plausible interior compositions for these planets^3–7^. These models generally assume a number of distinct interior layers and have exposed a degeneracy of interior compositions. This means that several interior models are compatible for a given mass and radius. There is no direct method to measure the interior composition of planets^8^. Available mass and radius data of exoplanets offer constraints on interior structures but do not yield unique solutions. In fact, there are many ways to derive plausible interior compositions^3,9–12^ and substantial degeneracies among interior compositions remain. Addressing the degeneracies requires an improved understanding of planetary materials at pressures and temperatures present within these interiors and may include additional assumptions about their formation and evolution. In particular, identifying conditions necessary for miscibility between layers is crucial to determine whether it is necessary to include a mixed layer and dilute or fuzzy cores in planetary interior models^13–18^. Furthermore, planet radii are sensitive to the water layer’s temperature profile^5,12^. We were compelled to study the interfaces between rock and icy material to investigate miscibility between the two and aid in developing more accurate and sophisticated planet models for water worlds.

The details of how materials accrete onto a planetary body and the evolution of planets into their present-day state are active research areas. Beyond the snow line, various silicates and volatiles comprise the reservoir of materials that can form these planets^19^. Some of the discovered super-Earths and sub-Neptunes are likely to be water-rich^20–23^. For example, Barth et al. suggested that the TRAPPIST-1 planets ${e}$, ${f}$, and ${g}$ may contain substantial amounts of water^15,17^. This work defines water worlds as planets of a rock and icy composition that do not contain a significant hydrogen atmosphere. Additional theoretical works propose water-rich exoplanets, with no atmosphere, can contain water mass fractions upwards of 37–50% ^6,24^. Typically, the super-Earths and sub-Neptunes that are hypothesized to be water worlds are assumed to be hotter than the water-rich bodies in our solar system due to their short orbital periods and proximity to their host star^8^. Therefore, the conditions at the rock-water boundary layer of these planets can reach substantially higher temperatures^25^. It is imperative to understand the role of miscibility on the internal structure of water worlds^16^. Identifying whether these chemical reactions occur between rock and water will offer great insight into a water world’s formation and evolution.

Models for the interiors of water worlds have assessed several magnesium silicates and water as plausible constituents of the interior^7,18,26,27^. The interaction between magnesium silicates and water depends on the planet’s formation history, and miscibility may occur at the surface level and deep within the planet. Understanding material interactions can give us insight into how these planets evolved into their present state. The extreme pressures and temperatures possible within the interiors of massive water worlds give rise to chemical interactions of rock and water not present at ambient conditions such as novel crystallographic phases, superionicity^28^, or the mixing of rock and water into an atomic, homogeneous mixture. We can also get insight into how and where water is incorporated in water worlds^7^, as well as their possible formation and evolution into their present state^15,17^. In addition, the *P*–*T* conditions reached during accretion by giant impacts reach TPa pressures and temperature of the order $10^4$ K, which implies the presence of a mixed layer with further enhanced water content. Planetary measurements of mass and radius are sensitive to water content due to its high compressibility^5,7,17^. A mixed layer would influence internal compositional profiles, the generation of magnetic fields, and the extent to which heat flows out of the planet. Understanding the chemistry and physics that occur at conditions pertinent to planetary interiors is required to characterize a planet’s dynamic evolution into their current structure.

**Figure 1:**
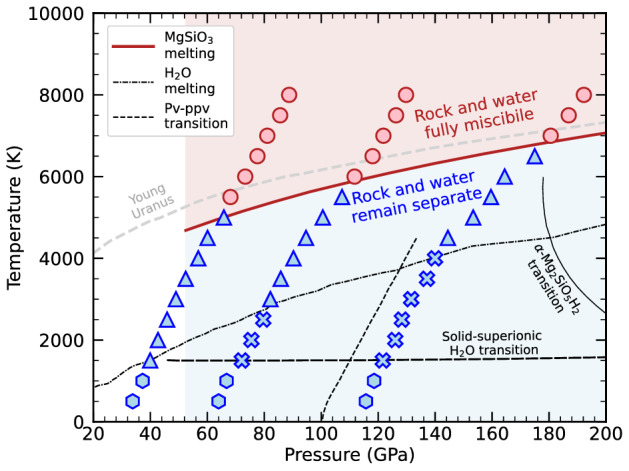
Isochores for systems 1-3 (starting from left to right) are plotted in *P*-*T* space. The blue symbols represent conditions under which rock and water remained separate while the red symbols indicate a fully mixed rock-water fluid. Our simulations predict that mixing occurs as soon as the rock melts. The *T*-*P* conditions agree with the experimental melting line of MgSiO$_3$^59^. With increasing temperature, water transitions from a solid (blue hexagons) to a superionic phase (blue crosses) and finally into a liquid state (blue triangles). The solid-superionic transition^28^ and H$_2$O melt curve^81^ are plotted. The pv-ppv phase transition was obtained from^82^. The transition to the ultra-high pressure phase $\alpha$-Mg$_2$SiO$_5$H$_2$ from Ref.^38^ is also plotted. The grey dashed line shows a model for a young Uranus planet at an age of 0.1 Gyr^16^.

The interaction between rocks and water has been extensively studied at low pressures ($< 1$ GPa) and temperatures relevant to Earth’s subduction zone^29^ as well as the properties of hydrous melts and peridotite-H$_2$O sytems^30–33^. However, at high pressures, water transforms into superionic phase at planetary interior conditions^34^ and silicates undergo a series of solid phase transformations. In the MgSiO$_3$ crystal structure, the silicon ions change from 4- to 6-fold coordinated with the oxygen ions at pressures of $\sim$ 55 GPa^35^. However, it has yet to be determined how these structural changes affect the chemical and miscibility properties at conditions in the interiors of water worlds. Recently, Kim et al.^18^ conducted high pressure experiments of Mg$_2$SiO$_4$ in H$_2$O in laser-heated diamond anvil cells and observed that magnesium oxide (MgO) leaches into water at 50 GPa and 1700 K, indicating that mixing of rock and ice is possible at elevated *P*–*T* conditions. Computational studies have investigated the properties of hydrous silicate melts, assuming rock and water are miscible at the lower mantle conditions^36,37^. Novel solid phases of Mg$_2$SiO$_5$H$_2$^38^ and (SiO$_2$)$_2$H$_2$O^39^ have also been predicted recently, using *ab initio* calculations, to form at very high pressures of $\sim$ 250 and 450 GPa. The stability field of the lower-pressure phase is shown in Fig. 1. A novel hydrous magnesium silicate phase has also been studied with experimental and computational methods^40–42^ and is predicted to be stable below $\sim$ 1500 K over a pressure range of approximately 42–60 GPa.

Assuming that rock and ice are fully miscible at all temperatures beyond the second critical point, Vazan et al.^16^ constructed interior models of water worlds with mixed layers. Similarly Dorn et al.^17^ assumed silicates and water were miscible at high pressure and used this assumption to compare the radii of planets with mixed interior layers with those of fully differentiated planets. It was determined that extended mixed layers can cause a radius reduction of several percent because water, the lighter and more compressible material, is exposed to higher pressure. Their work derived the density of rock-ice mixtures using the additive volume law (AVL). We will revisit this assumption with our calculations and confirm that the AVL is a very good approximation for predicting the density of MgSiO$_3$-H$_2$O mixtures.

In this work, we use density functional theory molecular dynamics (DFT-MD) to predict the miscibility of candidate materials at the *P*–*T* conditions relevant to water world interiors up to 200 GPa and 8000 K (see Fig. 1). We investigate bridgmanite and post-perovskite phases of MgSiO$_3$ and ice VIII and ice X phases of H$_2$O. The miscibility of high-pressure phases of MgSiO$_3$ and H$_2$O has not been studied experimentally and the interactions of these materials, are assumed, but unknown at extreme *P*-*T* conditions. In earlier works, DFT-MD has been used to determine miscibility of planetary material such as water in hydrogen and helium^14^ pertinent to Jupiter and of iron and MgO for terrestrial planets^13^. Therefore, we are motivated to use DFT-MD to evaluate a larger *P*–*T* landscape and extend petrological studies^6,18,43^ of rock and water interactions providing an upper bound for the miscibility curve of these mixtures at high pressures.

## Results

### Results from atomistic simulations

We simulated MgSiO${}_{3}$-H${}_{2}$O mixtures at conditions relevant to rock-water interfaces within water worlds. Three systems were simulated at different temperatures while keeping their densities constant. System 1: we prepared a superlattice of bridgmanite (MgSiO${}_{3}$) and ice VIII (H${}_{2}$O) at a density of 3.45 g cm$^{-3}$, corresponding to a pressure of 30 GPa at 0 K. System 2: we considered bridgmanite and ice X at a density of 3.85 g cm$^{-3}$(60 GPa); and system 3: post-perovskite and ice X at 4.38 g cm$^{-3}$(120 GPa) (see Fig. S5 in the supplementary material). Since the lattice constant mismatch between the MgSiO${}_{3}$ and H${}_{2}$O crystals is unavoidable, we strained the lattice constants of both structures to fit them in a single, periodic supercell. The relative change in the lattice constants of each crystal was less than 2.5%.

With our rock-water systems built, we step-wise increased the temperature from one DFT-MD simulation to the next in 500 K increments. Water transitioned through three phases during our calculations. Initially, it remained in the solid ice phase during the first couple temperature increments depicted by the blue hexagon symbols in Fig. 1. At temperatures beginning at $\sim$ 1500 K, water transitioned into the superionic phase where the oxygen atoms remain fixed in their lattice positions and the hydrogen atoms diffuse through that lattice^28^. We do not observe hydrogen atoms diffusing into the rock while the rock is solid and water is superionic (‘$\times$’ symbols in Fig. 1). At even higher temperatures, water melts completely while the rock remains solid and does not mix with water.

The temperature at which two materials become miscible can be determined with at least two different methods, in the same way, that melting temperatures of a material can be inferred: (1) Most simply, we can proceed in a similar manner to the heat-until-it-melts approach^44–47^ by performing a series of MD simulations at a fixed volume while increasing the temperature step by step until we observe mixing, which we will call heat-until-it-mixes approach. While the heat-until-melts approach is prone to overestimating the melting temperatures due to overheating of defect-free samples in computer simulations, our heat-until-it-mixes approach should not lead to a significant overestimation of this miscibility temperature because there are already a solid and a liquid phase present in our simulations that resembles the two-phase method^48^, which is regarded as an accurate method to obtain melting temperatures. Still, when the sample melts, the heat-until-it-melts method yields an upper bound to the melting point, and so it does our heat-until-it-mixes approach for the miscibility temperature. One should also note that there are cases when the heat-until-melts approach works very well. For example, the melting temperatures of beryllium predicted in this way are in good agreement with two-phase and free energy calculations^49^, and the solid-to-superionic transitions in water ice^28^ can be determined very accurately, where the transition can be reversed within a small temperature interval. (2) Alternatively, the melting temperature can be determined by computing the Gibbs free energies of both phases for a given pressure and temperature^28,49–53^. This is a much more complex process that typically involves some form of thermodynamic integration^13,14,54,55^. Similarly, one could determine the *P*-*T* conditions at which rock and water become miscible by comparing the Gibbs free energy of the mixture with that of the separate components.

In this paper, we determine the miscibility by pursuing the simpler heat-until-it-mixes approach. We expect this method to yield a reasonable upper bound of the true miscibility temperature because in our simulations, there are already a fluid and a solid phase present when the system transitions into fully mixed fluid state. We conducted our DFT-MD simulations step-wise from 500 to 8000 K in 500 K increments. If the two different materials spontaneously start to mix during our simulations, we know the materials are miscible at this temperature. With this comparatively simple method, we obtained novel results relevant to planetary science.

**Figure 2:**
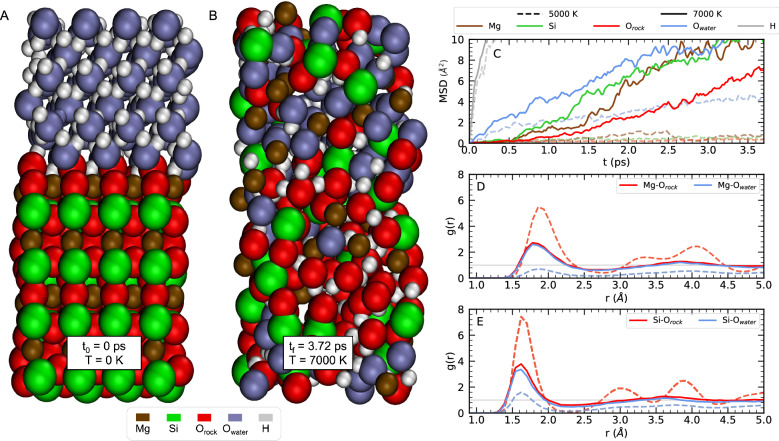
Simulations of system 3 relaxed to 120 GPa. (**A**): Initial configuration water (top) in the ice X phase and rock (bottom) in the ppv phase. (**B)**: Illustration of rocky-water miscibility at T = 7000 K obtained after a simulation time of 3.72 ps. In (**A**), (**B**) and (**C**), the atoms are colored by species: Mg (brown), Si (green), O$_{\text {rock}}$ (red), O$_{\text {water}}$ (blue), H (white). (**C**): Mean squared displacement in the vertical direction at 5000 and 7000 K as function of simulation time. Radial distribution functions between oxygen and magnesium or silicon atoms are shown in panels (**D**) and (**E**), respectively. Dashed lines represent the unmixed state (5000 K), while the solid lines illustrate a homogeneously mixed state (7000 K). In the unmixed state, the rock oxygen atoms are strongly correlated with nearby Mg and Si atoms (red dashed curves), while the water oxygen atom interactions only occur at the interface, where a couple Mg and Si atoms are nearby (blue dashed curves). In the mixed state, the distinction between oxygen atoms, which were present in the unmixed rock and water phases, disappears and the red and blue solid lines converge onto each other.

The most direct way to detect the melting and mixing of materials is by following the motion of the atoms throughout the trajectory. Once equilibrated, the mean squared displacement (MSD) was used to determine the onset of superionicity for water and distinguish between solid and liquid phases. However, while the MSD helps determine atomic diffusion, it does not show whether two materials are miscible because, while necessary, it is not a sufficient condition to determine homogeneous mixing. For example, once the water ice goes superionic, the MSD of hydrogen (H) atoms increases as a function of time which indicates diffusion, but does not indicate that there is mixing of rock and water. Similarly, if one layer melts, all of its atomic species will begin diffusing, the MSD values will increase over time, but the two fluid phases may remain separate. On the other hand, diffusion across the rock-water interface for all atomic species is a direct way to detect mixing (Fig. 2A, B). Therefore, we followed the motions of each atom in the direction perpendicular to the interface illustrated in Fig. 2C (and Figs. S2 and S3) to determine the mixing of material across the rock-water interface. It is straightforward to identify the transition to the mixed-phase with this method, and we employed it to derive the thermodynamic conditions that define the boundary between the miscible and immiscible regimes in Fig. 1.

Another method used to demonstrate material mixing is plotting the atomic radial distribution function (RDF). Figure 2D and E show how the Mg and Si nuclei are correlated with the oxygen atoms in the rock and water phases. For example, in the unmixed state at 5000 K (dashed curves in Fig. 2D and E) the Mg and Si atoms are strongly correlated with oxygen atoms in MgSiO$_3$, while they are less correlated with the oxygen atoms of H$_2$O, shown by the blue dashed curve. These correlations are similar to those found in silicate melts^37^ and pure MgO at much higher pressures and temperatures^56^, where a peak in the Mg-O *g*(*r*) function around 1.8 Å was also found for 2-fold compressed MgO and 5-fold compressed Mg^57^, which is larger than the Mg-O coordination distance for 2-fold compressed MgSiO$_3$^58^. However, under no conditions did we find a stable molecular bond between the Mg and O species when MgSiO_3_ is liquid. In the mixed state at 7000 K, the distinction between the groups of oxygen atoms disappears, and both types of oxygen atoms are equally correlated with the Mg and Si nuclei in a fluid mixture (solid lines). We see in Fig. 1 at temperatures above the MgSiO$_3$ melt curve^59^, rock and water fully become miscible. Therefore, we find that the MgSiO$_3$ melt curve is a lower bound for rock-water miscibility, but because we used the heat-until-it-mixes method, we may be overestimating the temperatures for miscibility. The melting of water before the rock should also result in depressed melting temperatures for rock and therefore lower the temperature for mixing. Nevertheless, using the heat-until-it-mixes approach, we show that rock-water miscibility occurs when rock melts and allows us to determine the conditions at which mixing occurs deep within water worlds. Therefore, these results suggest that these chemical interactions should be considered in models of planetary interiors.

### Results from impact simulations

During the rapid growth of planets, the combination of radiogenic heating and the heat of accretion leads to melting and separation into compositional layers. Nevertheless, these stratified layers can mix depending on the heat budget of the planet and the conditions reached within the interior, which can be influenced by giant impacts that deposit substantial energy deep inside a growing planet^60^. These major thermodynamic events transiently lead to a redistribution of material in an already differentiated body. Here, we consider whether collisions between water world planets could generate miscible layers of rock and water.

**Figure 3:**
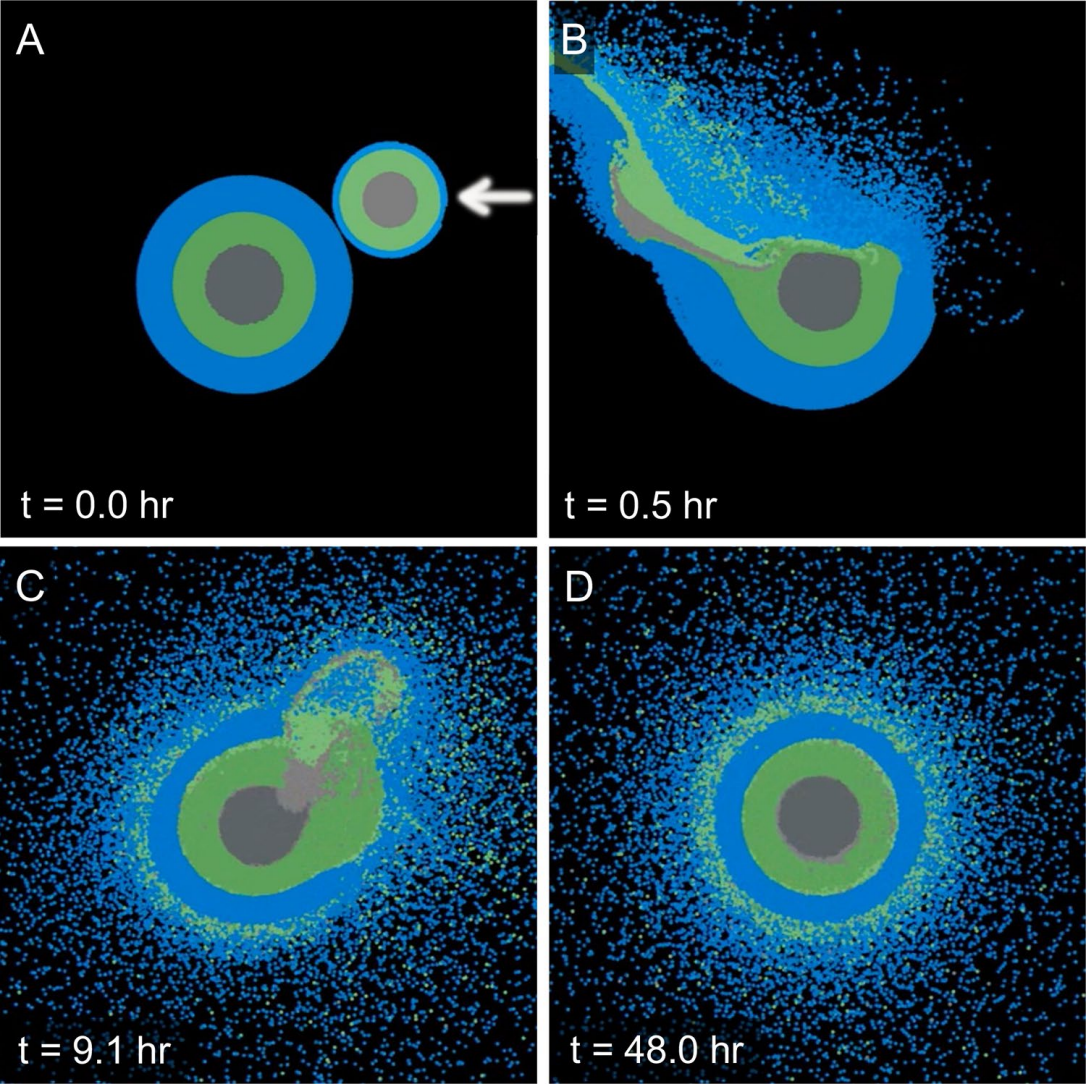
Equatorial slices of the material distribution during a giant impact graze-and-merger event. (**A**) 0.7 $M_{\oplus }$ body collides with a 4.7 M$_{\oplus }$ body at 23 km/s and a 30° angle as indicated by the white arrow (#3 in Table S3). Snapshots (**B**–**D**) show the time evolution after the impact in hours. The colors represent the materials: iron (grays), rock (greens), and water (blues).

We considered an exploratory set of accretionary giant impacts between 0.7 and 4.7 $M_{\oplus }$ mass planets with varying water fractions using a smoothed particle hydrodynamics (SPH) code (Tables S2 and S3). The final bodies have masses (3.8–5.3 $M_{\oplus }$) that fall near the observed transition in exoplanet mass-radius trends, around 1.5–2 Earth radii. In SPH codes, the continuum is represented by a large number of particles of fixed mass and composition, represented by an equation of state. Each body contained three distinct layers: metal core, silicate mantle, and pure water. As shown in Fig. 3, the material layers are shocked and mechanically mixed during a giant impact. Depending on the impact parameters and initial interior profiles, different amounts of each material are transiently mixed together. The treatment of materials in SPH does not enable consideration of miscibility, so during gravitational re-equilibration, the phases separate by buoyancy which tends to re-differentiate the merged body into strongly thermally stratified layers, as found in collisions between terrestrial planets with rock and iron layers^60^. In addition, some material is emplaced into orbit, and some escape the system. In general, during graze-and-merge collisions, the compositional layers are heated and partially mixed during the event (Movies S1 and S2).

**Figure 4:**
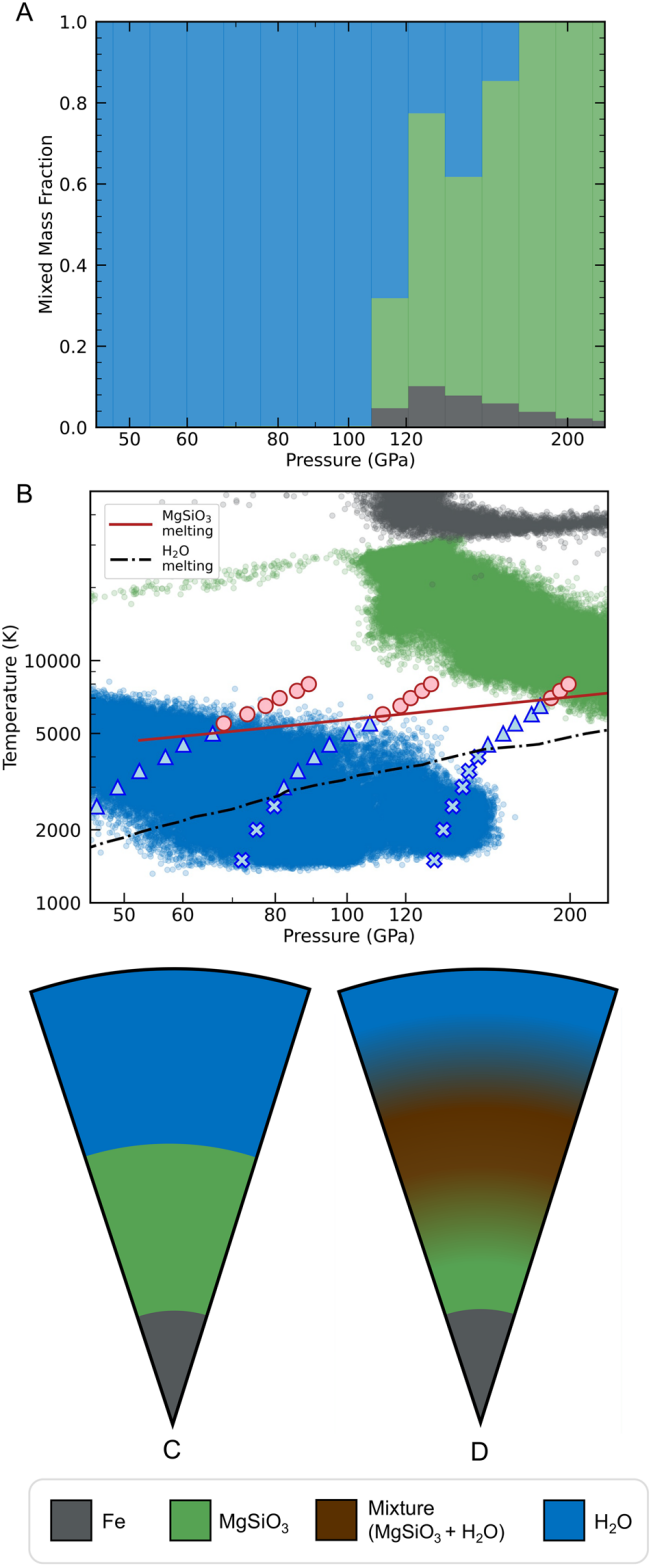
The top two plots show data points from the final frame of the giant graze-and-merge impact in Fig. 3D. The bar plot (**A**) shows the mass fractions of water (blue), rock (green), and iron (grey) as a function pressure on a logarithmic scale. We see a mass fractions of both rock and water across a pressure range from 110 to 190 GPa. In plot (**B**), the isochores from the DFT-MD simulations are overlaid onto the post-impact *P*-*T* data points of particles from the SPH simulation. Between the pressure ranges 110–180 GPa in plot (**B**) the rocky material is melted which is a criteria for mixing. Therefore, we conclude, based on the DFT-MD results, that there is a mixed rock-water region between 110 and 180 GPa of the water world post-impact. We include the melting curves of MgSiO$_3$^59^ (red line) and H$_{2}$O^81^ (black, dashed-dotted line) for reference. Below we show the pre-impact (**C**) and post-impact (**D**) interior profiles. (**D**) shows an extended, mixed layer of rock and water. (We do not include any mixing of iron and rock in this illustration.) The legend at the bottom of the figure shows material color assignments for (**A**), (**B**), (**C**) and (**D**).

After gravitational equilibration, the densities of the most shock-heated material may be lowered to a point where it is stable within an outer material layer, leading to pressure levels with mixed composition in the post-impact body, as shown in Figs. 4A and S5A. Here, super-heated metal is mixed into the silicate layer, and super-heated silicate is mixed into the water layer. The temperatures of these buoyant mantle particles are often much greater than the adjacent water particles, which correspond to the least shock-heated water (Figs. 4B, S5B). In an SPH calculation, this thermal stratification persists because of the absence of miscibility and heat transport. In a natural giant impact, thermal and chemical equilibration can occur within these mixed layers (brown shading in Fig. 4). When mixing occurs under conditions that exceed the miscibility boundary, the post-impact planet’s chemical profile contains a new compositional zone with a rock-water mixture.

In general, during accretionary giant impacts, we find that the outer portion of the silicate mantle is heated far above the melt curve temperature (Table S3) and partially mixed into the overlying water layer. For the cases explored here, 6–22% of the mantle material is mixed into the water layer at the end of the event. In each case, a greater portion of silicate experienced transient mixing with water during the collision, and in some cases, these transient contacts exceeded the miscibility threshold but could not be captured in the end state due to the limitations of SPH. In our calculations, the lower portions of the water layer sometimes reach the point of melting superionic ice, but exceeding this melt curve depends on the specifics of the initial planet (thermal profile and water layer thickness) and the impact conditions. In general, our results support the idea that when giant impacts occur between water worlds, conditions at the top of the rocky mantle exceed the conditions for miscibility. Even though much of the impact energy is deposited into the outer water layers, a large fraction of the internal energy increase is deposited in the deeper mantle materials. We found the internal energy increase of the mantles to correspond to 16–44% of the initial kinetic energy of the event (Table S3). How much water can dissolve into a mixed rock-water layer depends on the details of the event. Our results suggest that the mixed fraction of the rocky mantle can be substantial, on the order of 10% or more.

## Discussion

Previous works, e.g.^15,17^, have considered the dissolution of water into magma oceans on exoplanets by primarily focusing on lower pressure regimes found closer to the surface and rock-water interactions with an atmosphere rich in H$_2$O. Instead, we investigated rock-water miscibility at greater depths, or extreme pressures and temperatures, during the collisional growth of planets with a massive water layers overlaying a rocky interior. After giant impacts between water worlds, we expect their thermal evolution to be significantly influenced by the presence of a mixed, stably stratified compositional layer that evolves through secular cooling.

In Fig. 1, we also plot the adiabatic profile for a model ‘young’ Uranus with an age of 0.1 Gyr^8^. A substantial fraction of the heat from formation is still retained at this age, and the predicted *P*–*T* profile passes through our miscibility regime for rock and water. If ice-giant-type planets formed with such high interior temperatures (or experienced giant collisions as shown in Fig. 3), they should include an extended, mixed layer of rock and water in their interiors. Only after sufficient cooling will the rocky and icy components separate. The mixed layer may therefore be stratified and stable against convection^61^, delaying the planet’s cooling. Therefore, characterizing the miscibility properties of different planetary materials is vital in developing planetary evolution models across various planet types. As we discover and relearn how material interacts at extreme conditions, we need to shift our assumptions of fully differentiated planetary interiors to dynamic ones with ‘fuzzy’ interior layers where complex chemical interactions occur.

We studied the miscibility of rocky and icy material under conditions pertinent to water world interiors by performing DFT-MD simulations of bridgmanite and water mixtures at high pressure and temperatures. Under the conditions investigated, MgSiO$_3$ exists as either solid or liquid^59,62^ while H$_2$O can be solid, superionic, or liquid^28^. We find that rock and water are miscible and form a homogeneous mixture once MgSiO$_3$ melts. We computed the equation of state of the mixture and the validity of the AVL based on the equation of state of liquid MgSiO$_3$ and liquid H$_2$O at the same pressure and temperature conditions. Our findings indicate the predicted densities obtained from AVL are in excellent agreement with the density of the fully interacting mixture. For example, at 7000 K the AVL overpredicts density by only $0.3\%$.

We also performed *dynamic* smoothed particle hydrodynamics simulations of two colliding planetary bodies with a three-layer structure of metal, silicate, and water. We show that all three materials are sufficiently heated during impact events and become dynamically mixed. Therefore we provide proof for the presence of a mixed rock-water layer that is stably stratified and provided this layer remains sufficiently hot; it may persist for long periods during secular cooling. Since giant impacts are a ubiquitous stage of planet formation, our work suggests that the formation of miscible rock-water layers may be common in water-rich exoplanets. Studies of the internal properties of water worlds should consider the effects of mixing and demixing on their thermal evolution, and assumptions on non-ideal mixing should be revisited^63^. This work ultimately provides theoretical evidence that many water-rich exoplanets have mixed mantles.

## Methods

### DFT-MD

All density functional molecular dynamics (DFT-MD) simulations were performed using the Vienna Ab Initio Package (VASP) using the projector augmented-wave (PAW)^64–66^ method. The valence configurations for the atoms were Mg([Ne]3s$^2$), Si([Ne]3s$^2$3p$^2$), O([He]2s$^2$2p$^4$), and H(ultrasoft). A canonical ensemble (constant number of particles *N*, volume *V*, and temperature *T*) was used and temperature was regulated with the the Nosé-Hoover thermostat^67,68^. Exchange-correlation effects were modeled by the Perdew, Burke, and Ernzerhof functional^69^. All molecular dynamics simulations used a 0.2 fs timestep. Electronic wave functions were expanded in a plane-wave basis with an energy cut-off of 1400 eV and we used the $\Gamma$-point to sample the Brillouin zone of our supercells for all simulations.

The supercells consisted of MgSiO$_3$ and H$_2$O merged along the [001] direction (see Figs. 2A and S6). The crystal phases used for MgSiO$_3$ were bridgmanite (30 GPa and 60 GPa) and post-perovskite (120 GPa). The phases for water used were ice VIII (30 GPa) and ice X (60 and 120 GPa). System 1 (bridgmanite-ice VIII) refers to the initial 0 K configuration of the rock-water cell at 30 GPa, system 2 (bridgmanite-ice X) the 0 K configuration of the rock-water cell at 60 GPa, and finally system 3 (ppv-ice X) being the 0 K configuration of the rock-water cell at 120 GPa. The water to rock ratio is defined as the number of H$_2$O formula units over the total number of MgSiO$_3$ and H$_2$O formula units in the system. Therefore, system 1 has 32 MgSiO$_3$ units and 64 H$_2$O units resulting in an water to rock ratio of 0.26. System 2 and 3 have 32 MgSiO$_3$ units and 72 H$_2$O units leading to a water to rock ratio of 0.29. System size convergence tests were performed to verify that these cells provide sufficiently well-converged results. The details are provided in the supplementary material.

In system 3, the orthorhombic primitive cell for MgSiO$_3$ was replicated $4\times 1\times 1$, resulting in cell dimensions of $9.9098 \times 8.1253 \times 6.1493$ Å$^{3}$. For the ice X phase, the cubic primitive cell was replicated $4\times 3\times 3$, leading cell lengths of $10.4738 \times 7.8554 \times 7.8554$ Å$^3$. Since the lattice constant mismatch between the MgSiO$_3$ and H$_2$O crystals is unavoidable, we strained the lattice constants of both structures to fit them in a single, periodic supercell. The cell dimensions for system 3 resulted in a superlattice of dimensions $10.1920\times 7.9900\times 14.6046$ Å$^3$. Since the lattice constants were strained, we performed an additional ionic relaxation within the supercell to remove residual stresses. The lattice constants of the supercell were relaxed again to achieve the hydrostatic conditions at a pressure of 120 GPa. The dimensions of this supercell were $9.8191\times 8.0286\times 15.1232$ Å. This was used as the initial configuration for the DFT-MD simulations, for system 3 in particular, and we equilibrated it at temperatures ranging between 500 and 8000 K, in 500 K degree steps.

When the MD trajectories were equilibrated, analysis to determine miscibility was performed. The mean squared displacement (MSD) in the z-direction (perpendicular to the interface), given by

1 $$\begin{aligned} \mathrm{MSD}_\alpha (t) = \left<( z(t) - z_0 )^2 \right>_{i,\alpha } = 2D_{\alpha }t \end{aligned}$$

where $<>_{i,\alpha }$ denotes a statistical average over all particles of type $\alpha$ and $D_\alpha$ the diffusion coefficient, was used to determine the onset of superionicity in the ice and distinguish a solid from liquid. When $D_\alpha >0$ for all species, the sample is liquid, while $D_{\mathrm{H}}>0$ with $D_\alpha =0$ for all other species represents superionic ice. We found that this method helped determine atomic diffusion but could not tell us whether the materials were miscible, as diffusion is a necessary but not sufficient condition to generate a homogeneous mixture.

Therefore, we also rely on the analysis on the individual trajectories of the atoms to confirm that the atomic species diffuse through the system instead of the two separate liquid compounds remaining. We calculated the radial distribution function for each atomic species using,

2 $$\begin{aligned} {\mathrm{g}}_{\mathrm{ab}}(r) = \frac{1}{4\pi r^{2} {{\text {N}}_{\alpha }} {{\text {N}}_{\beta }}} \sum _{i=1}^{{\mathrm{N}}_{\alpha }} \sum _{j=1}^{{\mathrm{N}}_{\beta }} \left\langle \delta ( \Vert {\mathbf {r}}_i^\alpha - {\mathbf {r}}_j^\beta \Vert - {\mathrm{r}}) \right\rangle \end{aligned}$$

where we calculate the distances between two atomic species of interest $\alpha$ and $\beta$ throughout a trajectory. $\delta (r)$ is the Dirac delta function. The prefactor guarantees that the function equals 1 for noninteracting particles.

### SPH

The GADGET-2 smoothed particle hydrodynamics (SPH) code^70^, modified to include tabulated equations of state (EOS)^71^, was used to calculate the structure of isolated bodies and to simulate giant impacts. The giant impact simulations were conducted in a similar manner as in previous work^72^, and the source code is available in the supplementary information in^72^ and https://github.com/PhilJCarter/gadget2-planetary. Here, we used an updated version of the Analytic Equations of State (ANEOS) code package that produces a better fit to shock and post-shock temperatures^73,74^. The source code for ANEOS is available in^75^.

For this initial study, we considered water worlds with three distinct compositional layers: metal, silicate, and water. As the properties of water world interiors are largely unknown, the pre-impact bodies were assumed to be analogous to terrestrial bodies with a solid silicate mantle and liquid iron-alloy core. The thermal profile of the mantle was chosen to fall near the solidus of peridotite at the mantle-water boundary, which is a conservatively cool initial condition during the giant impact stage of planet formation. All had a metal core to silicate mass ratio comparable to Earth’s value of 0.32. The planets had an exploratory range of initial water entropies, and the total water mass fractions ranged from 7wt% to 50wt%. The metal, silicate, and water layers were modeled using ANEOS models for an iron alloy (Fe$_{85}$Si$_{15}$)^76^, a simplified pyrolitic composition (Ca$_{0.87}$Fe$_{2.03}$Mg$_{20.22}$Al$_{1.98}$Si$_{16.27}$O$_{58.63}$), and pure water, respectively. Currently, there is no ANEOS model available for MgSiO$_3$, and we used a new pyrolite ANEOS, a model composition for the bulk silicate Earth^77^ that is a modification of the forsterite (Mg$_2$SiO$_4$) ANEOS model^73^ but with improvements to the liquid phase. The water ANEOS model is a new parameter set that takes advantage of the triatomic formulation for the gas and improves the model vapor curve compared to previous versions. The ANEOS formulation treats each material as a single component with solid, liquid, vapor and plasma phases. Each of these natural materials has a more complicated phase diagram with multiple solid structures and, in the case of pyrolite, a multi-component composition. The EOS models are developed to capture the bulk response of each material. The ANEOS material parameters are given in Table S1 and further documentation are available at https://github.com/ststewart. Non-rotating, isolated bodies were initialized with a specified mass and entropy for each material layer (Table S2). The bodies were then gravitationally equilibrated for 24 hours. At each time step, the entropy of each layer was imposed and the particle velocity was damped, reaching residual velocities of 50-60 cm s$^{-1}$.

Here, we considered giant impacts that are typical of accretionary events between protoplanets^78,79^. The most common growth events were graze-and-merge collisions with intermediate impact angles. More grazing impact angles (e.g., $\gtrapprox 45^{\circ }$) lead to hit-and-run events. Simulations were typically run for 48 hours to achieve gravitational re-equilibration, but some required longer times. In most cases, the graze-and-merge events created synestias^80^ with massive water-rich disk regions. The impact parameters and post-impact properties for the water world giant impacts are presented in Table S3. For all cases considered, a substantial fraction of the mantle (6-20%) is super-heated and mixed into the outer water layer. Generally, the temperatures of the silicate at the pressures of the mantle-water interface substantially exceed the melt boundary and promotes miscibility with water.

## Supplementary Information

Supplementary Information 1.

Supplementary Information 2.

Supplementary Information 3.

Supplementary Information 4.

Supplementary Information 5.

Supplementary Information 6.

Supplementary Information 7.

## Data Availability

All DFT-MD data are included in the manuscript and in the SI Appendix. SPH simulation initial and final timestep files will be deposited in Dataverse upon acceptance of this manuscript and linked here.
