# Supplementary material for: Miscibility of rock and ice in the interiors of water worlds
Source: Sci Rep. 2022 Jul 29;12:13055. doi: 10.1038/s41598-022-16816-w (PMC9338078; doi:10.1038/s41598-022-16816-w)
Supplement: Supplementary file 4 — Supplementary Information 4. [file 41598_2022_16816_MOESM4_ESM.pdf]

Supplementary Information for

Miscibility of Rock and Ice in the Interiors of Water Worlds

Tanja Kovačević, Felipe González-Cataldo, Sarah T. Stewart, Burkhard Militzer

Corresponding Author Name: Tanja Kovačević
E-mail: tanja_kovacevic@berkeley.edu

This PDF file includes:
Supplementary Figures S1 to S6
Supplementary Tables S1 to S3
Legend for Movies S1 to S3
Legends for Datasets S1 to S3
SI References

Supplementary text 1. Finite Size Effects

We converged our simulations with respect to system size by generating simulation cells of different sizes and performing DFT-MD simulations of miscibility. First, we built a 4x1x1 Ppv supercell with 16 $MgSiO_3$ formula units that we fit together with a 4x3x3 ice-X supercell with 72 H_2O formula units in a single supercell of 296 atoms, as shown in the figure below. We performed DFT-MD simulations and found that the transition to superionic ice occurred at 1500 K, in agreement with previous studies [1]. However, we found that $MgSiO_3$, in the rock-ice supercell S1(A), melted at temperatures well below the empirically predicted melting line for pure $MgSiO_3$ [2,3]. We attributed this discrepancy to size effects. We decided to add additional replicas of the Ppv primitive cell in the [001] direction, increasing the amount of $MgSiO_3$ formula units from 16 Fig. S1(A), 32 Fig. S1(B), and 48 Fig. S1(C). The supercells (B) and (C) reach the liquidus and mix at the same temperature step given our step size of 500 K. The miscibility of rock and ice, in supercells (B) and (C), occurs once rock melts. The melting of rock in our simulations are in good agreement with the melting line of $MgSiO_3$[2]. Therefore, the supercell (B) with 376 atoms, S1(B), is a sufficient cell size.

In order to address the size of the cross section area, we would need to find a commensurate number of replicas for both H_2O and $MgSiO_3$ supercells, such that they both fit in the same supercell requiring only small strains. However, the next available supercell following S1(A) would require $8\times3\times3$ for H_2O and $8\times1\times1$ for $MgSiO_3$, consisting of 592 atoms S1(D), which is beyond our simulation capabilities. Obtaining a one picosecond trajectory for this large supercell (S1(D)) would require 25 days of simulation. Increasing the cross section area of S1(B) would drastically increase simulation times.

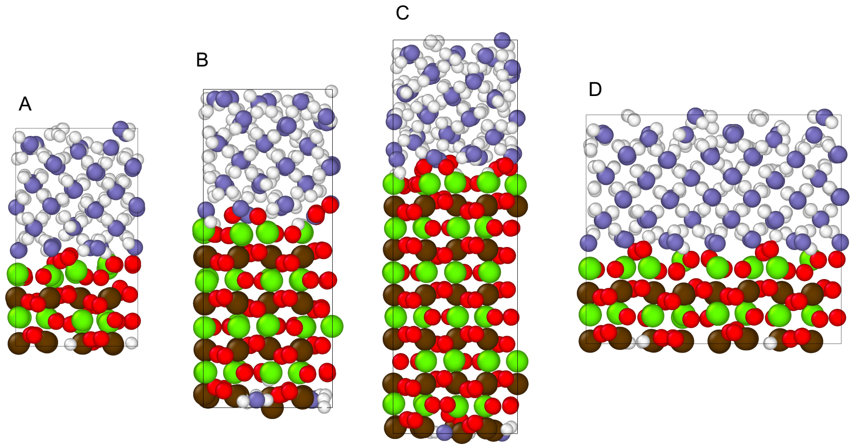

Supplementary Figure S 1. Visualizations of the simulation cells used to study size-effects: (A) 16 $MgSiO_3$ formula units and 72 H_2O formula units, (B) 32 $MgSiO_3$ formula units and 72 H_2O formula units, (C) 48 $MgSiO_3$ formula units and 72 H_2O formula units, (D) 32 $MgSiO_3$ formula units and 144 H_2O formula units. The simulation cell in (B) is the supercell used for System 2, shown also in Fig. S6(b) below.

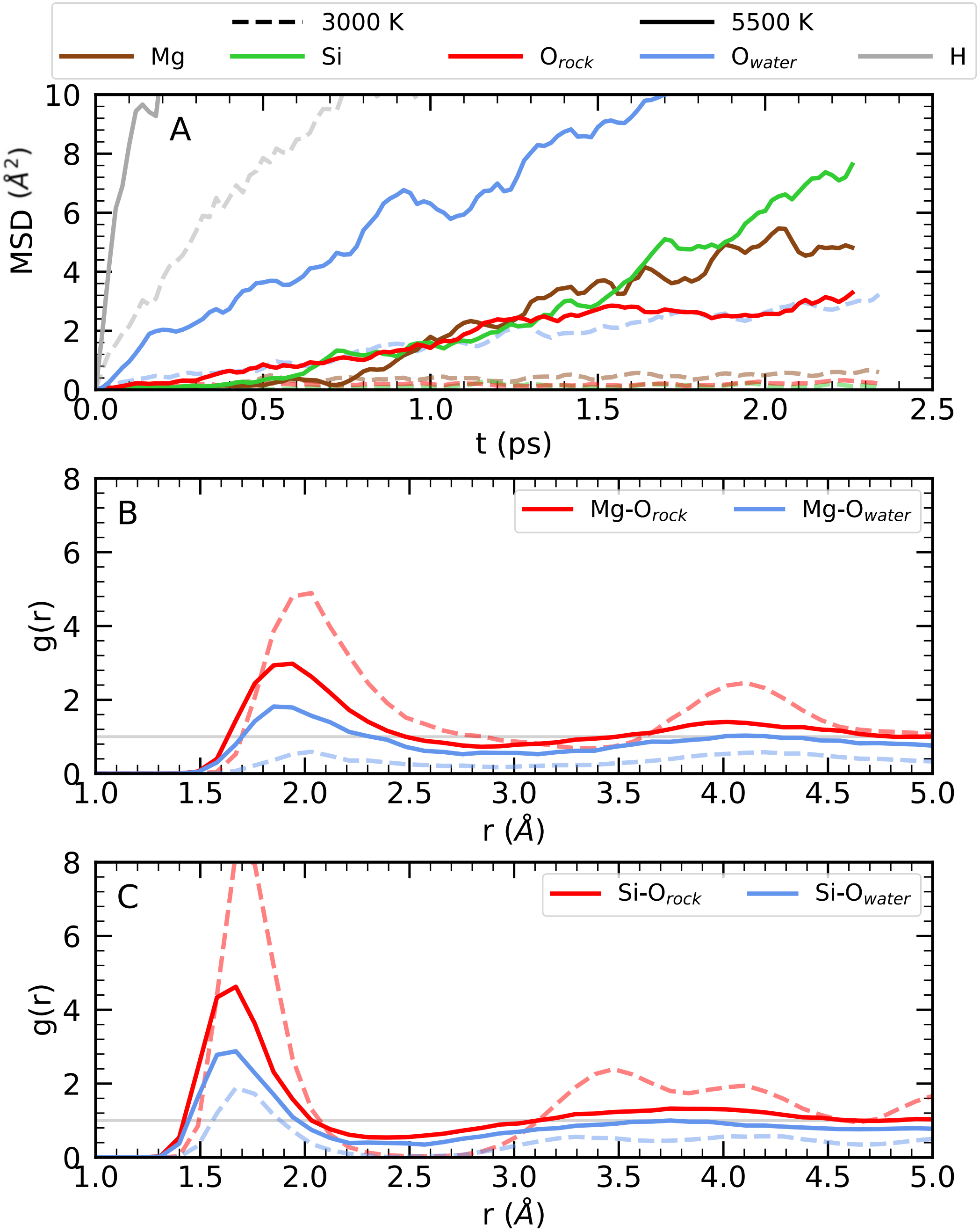

Supplementary Figure S 2. MSD and RDFs of system 1 (30 GPa). **A**: Atoms are colored by species: Mg (brown), Si (green), O_{rock} (red), O_{water} (blue), H (white). The mean squared displacement in the vertical direction at 3000 and 5500 K as function of simulation time. Radial distribution functions between oxygen and magnesium or silicon atoms are shown in panels **D** and **E**, respectively. Dashed lines represent the unmixed state (3000 K), while the solid lines illustrate a homogeneously mixed state (5500 K). In the unmixed state, the rock oxygen atoms are strongly correlated with nearby Mg and Si atoms (red dashed curves), while the water oxygen atom interactions only occur at the interface where a couple Mg and Si atoms are nearby (blue dashed curves). In the mixed state, the distinction between oxygen atoms, which were present in the unmixed rock and water phases, disappears and the red and blue solid lines converge onto each other.

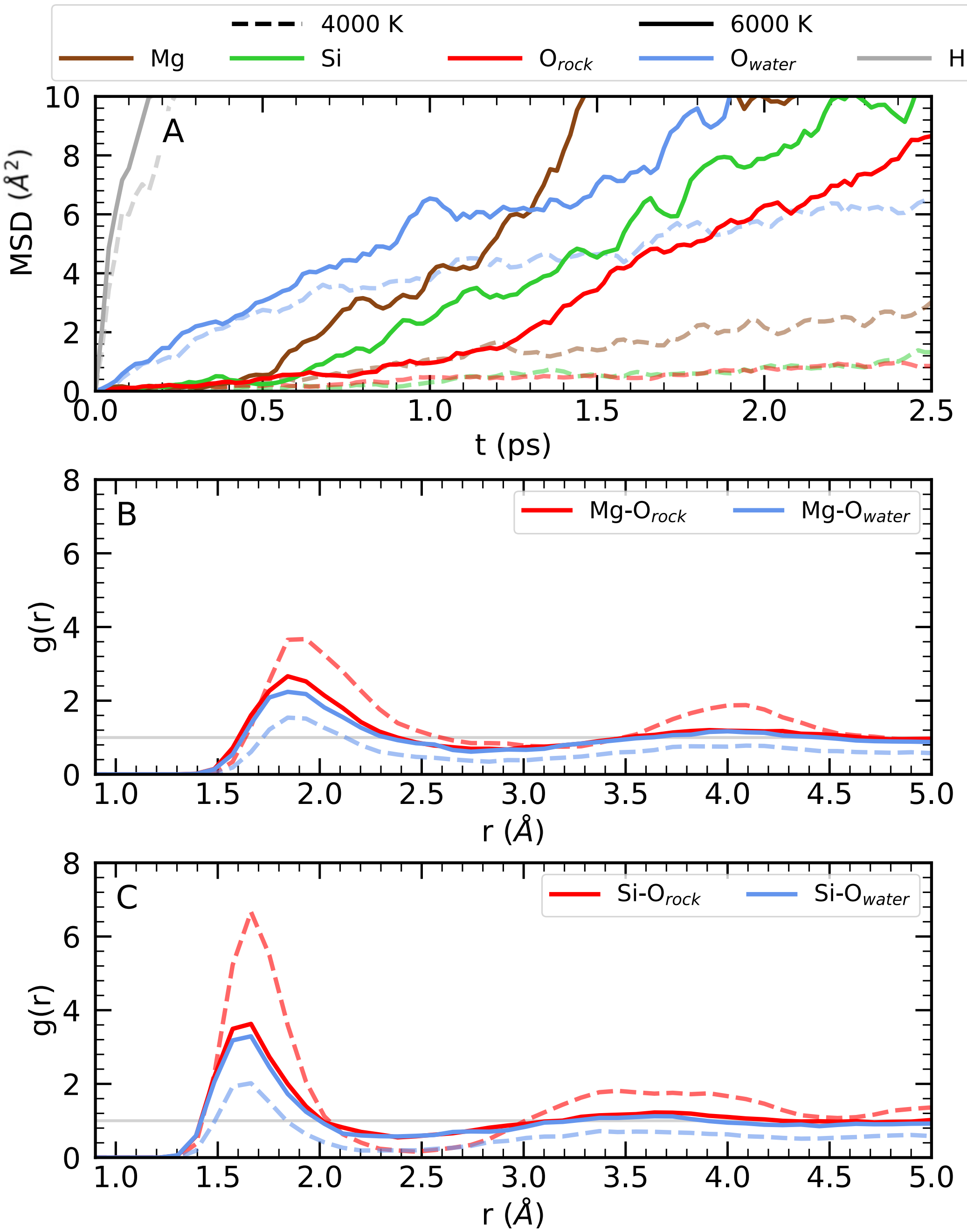

Supplementary Figure S 3. MSD and RDFs of system 2 (60 GPa). **A**: Atoms are colored again by species: Mg (brown), Si (green), O_{rock} (red), O_{water} (blue), H (white). The mean squared displacement in the vertical direction at 4000 and 6000 K as function of simulation time. Radial distribution functions between oxygen and magnesium or silicon atoms are shown in panels **D** and **E**, respectively. Dashed lines represent the unmixed state (4000 K), while the solid lines illustrate a homogeneously mixed state (6000 K). In the unmixed state, the rock oxygen atoms are strongly correlated with nearby Mg and Si atoms (red dashed curves), while the water oxygen atom interactions only occur at the interface where a couple Mg and Si atoms are nearby (blue dashed curves). In the mixed state, the distinction between oxygen atoms, which were present in the unmixed rock and water phases, disappears and the red and blue solid lines converge onto each other.

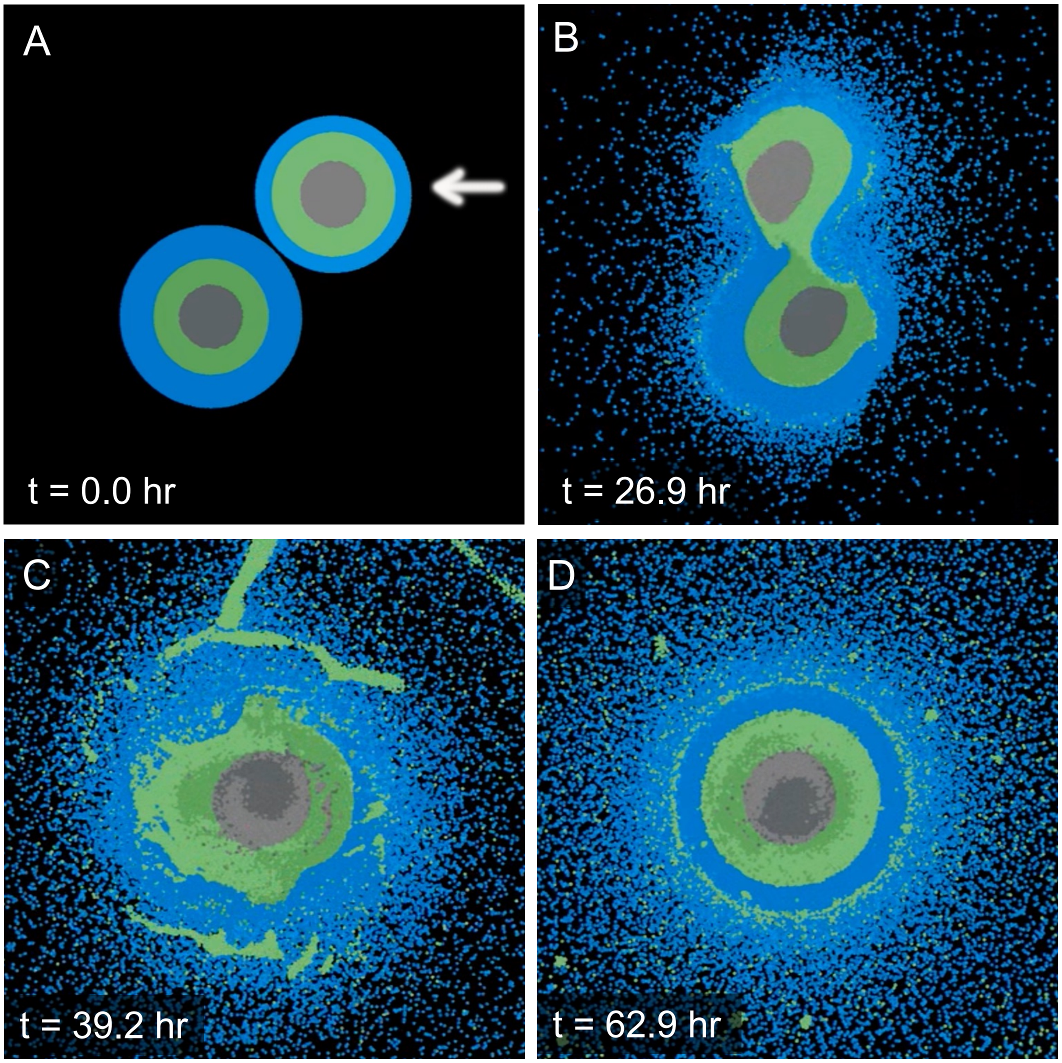

Supplementary Figure S 4. Equatorial slices of the material distribution during a giant impact graze-and-merge event. **A** M_{targ} = 2.96, $M_{\oplus}$, M_{proj} = 2.23 $M_{\oplus}$, V = 15.8 km/s, b = 0.7071 (Sim.#4 in Table S3). Snapshots **B-D** show the time evolution of the giant impact. The planetary material is color coded as follows: iron (grays), rock (greens), and water (blues).

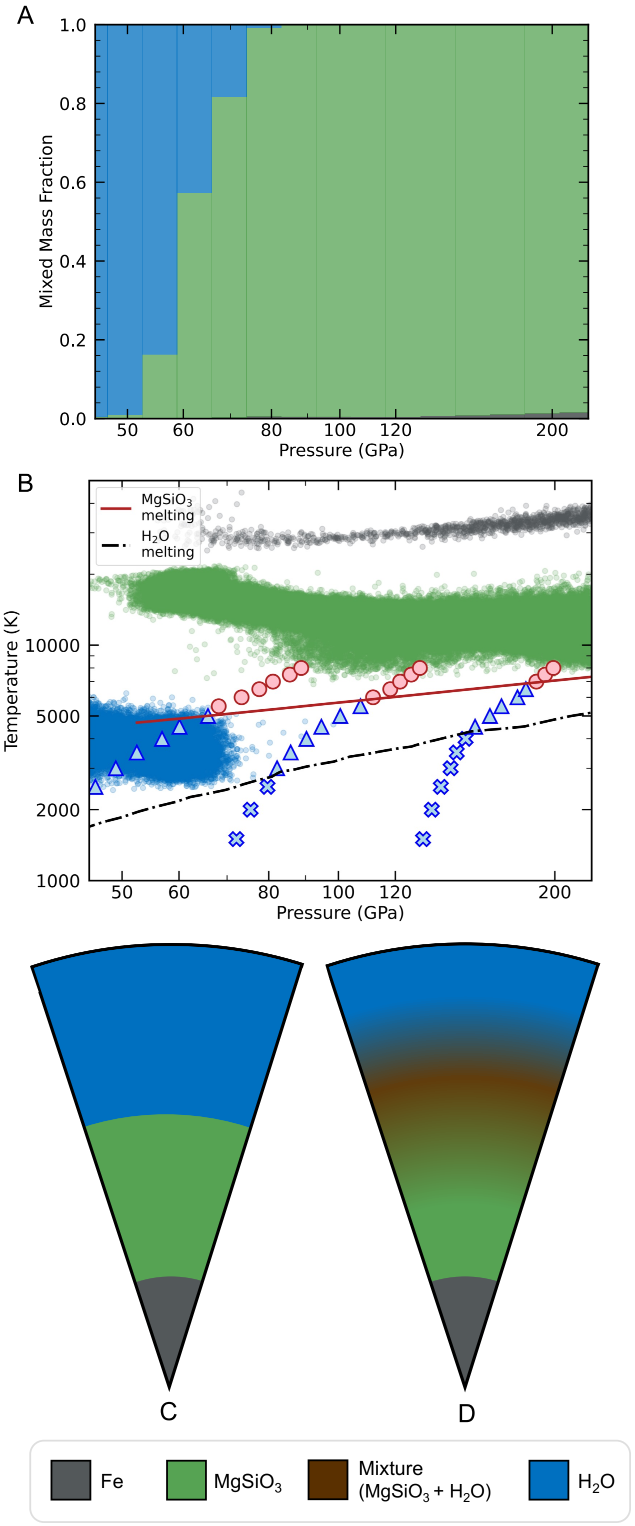

Supplementary Figure S 5. The top two plots show data points from the final frame of the giant graze-and-merge impact in Fig.**S3**. The bar plot **A** shows the mass fractions of water (blue), rock (green), and iron (grey) as a function pressure on a logarithmic scale. We see a ratio of rock and water across a pressure range from 50 – 80 GPa. In plot **B**, the isochores from the DFT-MD simulations are overlaid onto the post-impact P-T data points of particles from the SPH simulation. We include the melting curves of $MgSiO_3$[2] (red line) and H_2O[4] (black, dashed-dotted line) for reference. Below we show the pre-impact (**C**) and post-impact (**D**) interior profiles. **D** shows an extended, mixed layer of rock and water. (We do not include any mixing of iron and rock in this illustration.) The legend at the bottom of the figure shows material color assignments for **A, B, C,** and **D**.

Supplementary Table S 1. Equation of State Material Parameters using ANEOS.

Parameter	Definition	$Fe_{85}Si_{15}$	Pyrolite	Water
V01 N_{elem}	Number of elements	2	6	2
V02 EOS Type	4 is solid-liquid-gas with ionization	4	4	4
V03 ρ_0	Reference density, g cm^{-3}	7.51	3.35	1.25
V04 T_0	Reference temperature, K	298	298	150
V05 P_0	Reference pressure, dynes cm^{-2}	1E6	1E6	1E6
V06 B_0	Bulk modulus, dynes cm^{-2}	1.51E12	0.95E12	2.7E10
V07 γ_0	Grüneisen parameter at reference state	2.0	0.85	0.60
V08 θ_0	Debye temperature, K	-600	-1500	-290
V09 t	Cold curve model parameter $T_\gamma = t - 1$	0	1	1
V10 $3\gamma_{\text{inf}}$	Grüneisen parameter as $\rho \to \infty$	1.5	4.5	6
V11 E_{sep}	Zero temperature separation energy, erg g^{-1}	10.2E10	1.9E11	2.9E10
V12 T_{melt}	Melting temperature at reference pressure, K	1600	2163	273
V13 $C53$	Critical point adjustment parameter, erg g^{-1}	0	0	0
V14 $C54$	Critical point adjustment parameter	0	0	0
V15-V16	Thermal conductivity parameters, not used	0	0	0
V17 ρ_{min}	Minimum density for solid, default $0.8\rho_0$	0	0	0
V18-V22	High-pressure phase transition parameters, not used	0	0	0
V23 H_{fusion}	Enthalpy of fusion, erg g^{-1}	2.47E9	1.45E10	1.4E9
V24 ρ_l/ρ_s	Volume change on melting	0.962	0.90	0.89
V25 Upper	Upper limit to cold curve extension, default 1	0	0	0
V26 Lower	Lower limit to cold curve extension, default 0	0	0	0
V27 α	Liquid model parameter ($0 < \alpha < 1$, default=0.3)	0.3	0.3	0.05
V28 β	Liquid model parameter ($0 < \beta < 1$, default=0.1)	0.1	0.1	0.1
V28 γ	Liquid model parameter ($0 < \gamma < 1$, default=0.2)	0.2	0.2	0.2
V30 $C60$	Grüneisen model adjustment parameter	0	0	0
V31 $C61$	Grüneisen model adjustment parameter ($-1 < C61 < 0$)	-0.75	-0.80	-0.4
V32 $C62$	Critical point adjustment parameter ($0 < C62 < 1$)	0.5	0.5	0.5
V33 Flag	Ionization model, 0=Saha, 1=Thomas-Fermi	0	0	0
V34-V35	Reactive chemistry model parameters, not used	0	0	0
V36 N_{atom}	Number of atoms in molecular clusters	0	2	3
V37 E_{bind}	Molecular cluster binding energy, eV	0	4.25	4.67
V38 RotDOF	Rotational degrees of freedom	0	2	3
V39 R_{bond}	Length of molecular bond, cm	0	1.5E-8	96E-10
V40 VibDOF	Vibrational degrees of freedom	0	1	3
V41 T_{Debye}	Vibrational Debye temperature, K	0	2000	2000
V42 LJ Flag	Flag for Lennard-Jones (1) or Morse (0) potential	0	1	1
V43 a_{exp}	Exponent in Lennard-Jones potential	0	1.70	1.5
V44 f_{cv}	Thermal model adjustment parameter, max. $c_v = 3 f_{cv} NkT$	1.33	1.37	1.
V45 $QCC1$	Density to transition to ideal gas, g cm^{-3}	1E-20	1E-30	1E-30
V46 $QCC6$	ψ value to transition to ideal gas	1E5	1E5	1E5
	Atomic Numbers	14	8	1
		26	12	8
			13	
			14	
			20	
			26	
	Atomic Fractions	0.15	0.5863	0.6667
		0.85	0.2022	0.3333
			0.0198	
			0.1627	
			0.0087	
			0.0203	
	EOS table version code	SLVTv0.2G1	SLVTv0.2G1	SLVTv0.3G1

This work used ANEOS code package version 1.0[5]. Note input values of 0 sometimes flags the use of a default value. V08: Negative means use full Debye model; positive means use high-temperature approximation; T_{Debye} does not represent the true Debye temperature of the solid when $f_{cv} \neq 1$. V36-43: parameters for molecular gas developed by[?] and extended to triatomic molecules[5]. V44: thermal model adjustment parameter to fit liquid shock temperatures f_{cv}[6]. V45-V46: user adjustments to ideal gas transition[7].

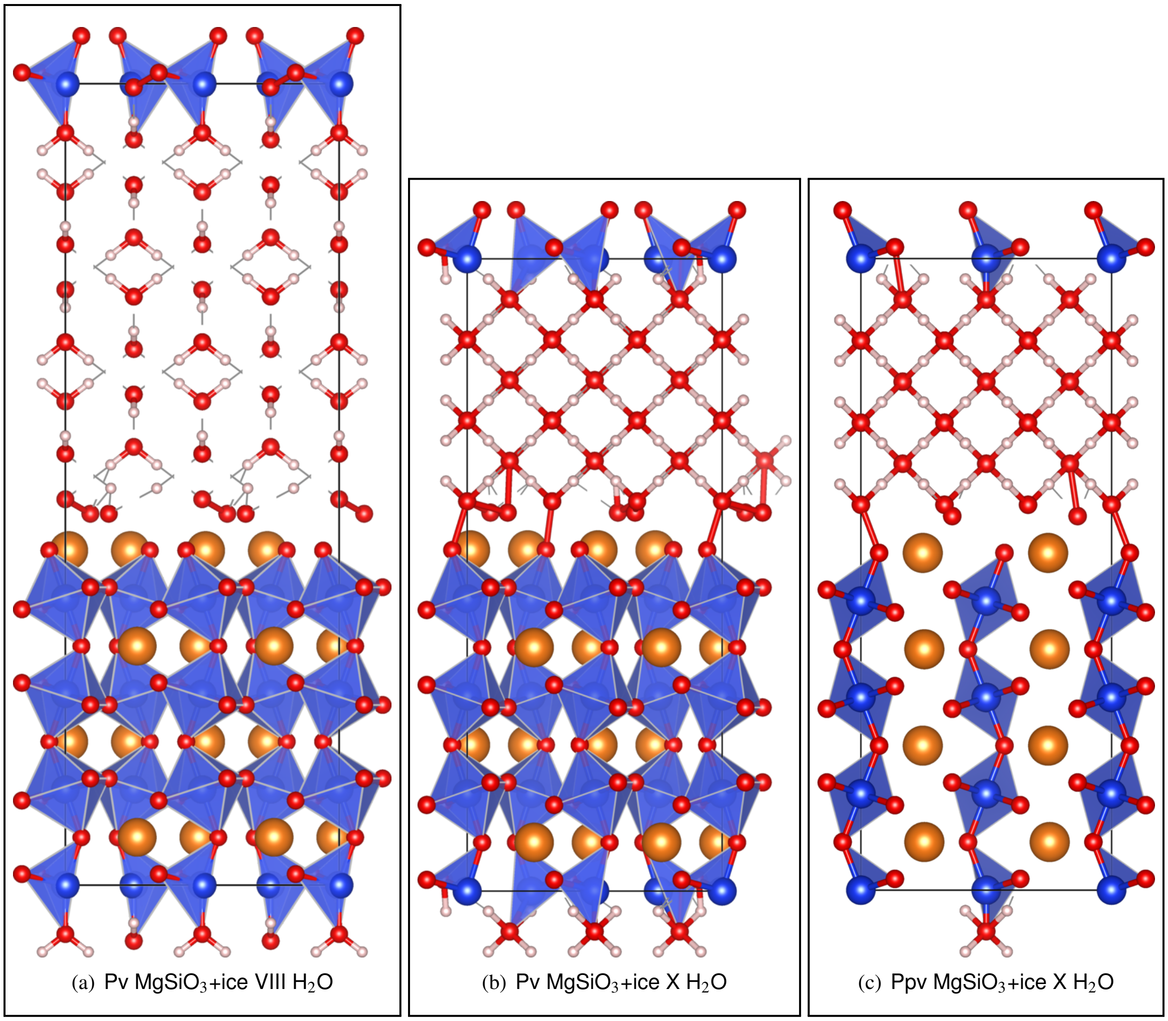

Supplementary Figure S 6. System 1(a): bridgmanite and ice VIII at 30 GPa. System 2(b): bridgmanite and ice X at 60 GPa. System 3(c): post-perovskite $MgSiO_3$ and ice X at 120 GPa. The space group for each crystal phase are as follows: Pv space group *Pnma*, Ppv space group *Cmcm*, ice VIII space group $I4_1/amd$, ice X space group *Pn-3m*. The corresponding densities are 3.1560, 4.3585, 4.4156 $g\,cm^{-3}$.

Supplementary Table S 2. Summary of initial conditions for water world SPH planets.

M_{tot} [$M_\oplus$]	M_{core} [M_{tot}]	M_{mantle} [M_{tot}]	M_{water} [M_{tot}]	N_{part} [#]	S_{water} [kJ/K/kg]	S_{mantle} [kJ/K/kg]	S_{core} [kJ/K/kg]	R [km]	V_{esc} [km/s]
0.70	0.27	0.57	0.15	70000	3.0	2.70	1.79	6350	9.4
1.94	0.23	0.50	0.27	194000	9.0	2.95	1.81	9220	12.8
2.13	0.27	0.66	0.07	213000	3.0	2.70	1.79	8440	14.2
2.23	0.23	0.52	0.25	223000	6.0	2.85	1.81	9240	13.9
2.96	0.16	0.34	0.50	295750	3.0	3.10	1.79	10730	14.9
4.68	0.18	0.38	0.44	468000	3.0	3.20	1.79	11880	17.7

M: total mass and masses of each material layer; N_{part}: number of SPH particles; S: specific entropies of each material layer; R: initial radius (±10 km) based on 1-D interior model calculation (not dependent on N_{part}); V_{esc}: escape velocity based on total mass and R.

Supplementary Table S 3. Summary of giant impact simulations.

Sim. #	$M_{\rm targ}$ $[M_\oplus]$	$M_{\rm proj}$ $[M_\oplus]$	$V_{\rm i}$ [km/s]	$V_{\rm i}$ $[V_{\rm esc}]$	b -	x_0 -	Q_S [MJ/kg]	$M_{\rm bnd}$ $[M_\oplus]$	$M_{\rm tr}$ $[M_\oplus]$	$R_{\rm tr}$ [Mm]	Mass frac. C/M/W	$P_{\rm mw}$ [GPa]	$R_{\rm mw}$ [Mm]	$T_{\rm m}$ [K]	$T_{\rm w}$ [K]	dIE/KE$_0$ C/M/W	$M_{\rm mix,m}$ $[M_{\rm bnd,m}]$
1	4.68	0.70	16.9	1.1	0.707	0.7	2.3	5.2	0.14	19.06	0.20/0.41/0.40	137	8.46	28200	2400	0.08/0.23/0.32	0.14
2	4.68	0.70	16.9	1.1	0.500	0.9	7.7	5.3	0.13	17.29	0.19/0.41/0.40	130	8.66	28400	2400	0.10/0.30/0.38	0.11
3	4.68	0.70	23.0	1.5	0.500	0.9	14.2	5.1	0.20	18.02	0.20/0.41/0.38	123	8.42	25300	2600	0.05/0.16/0.23	0.16
4	2.96	2.23	15.8	1.1	0.707	0.7	5.5	5.1	0.72	14.46	0.19/0.42/0.38	59	10.06	18300	4100	0.06/0.18/0.22	0.11
5	2.96	2.23	15.8	1.1	0.500	0.9	18.5	5.1	0.72	13.11	0.19/0.42/0.39	69	9.85	20200	4500	0.09/0.23/0.31	0.13
6	2.23	2.23	14.0	1.0	0.500	0.9	16.3	4.4	0.47	12.67	0.23/0.52/0.24	38	10.34	15600	4900	0.12/0.30/0.27	0.10
7	2.23	2.23	17.4	1.3	0.500	0.9	25.2	4.3	0.69	13.89	0.24/0.54/0.22	24	10.75	13300	5200	0.06/0.21/0.15	0.20
8	2.23	1.94	13.5	1.0	0.707	0.9	4.1	4.1	0.69	11.48	0.23/0.52/0.25	22	11.10	12100	3800	0.09/0.23/0.21	0.09
9	2.23	1.94	16.8	1.3	0.500	0.9	21.5	4.0	0.67	12.85	0.24/0.53/0.23	11	11.44	12900	6600	0.06/0.21/0.16	0.12
10	2.14	2.14	17.8	1.3	0.500	0.9	26.4	4.1	0.60	12.87	0.28/0.67/0.05	7	12.08	13300	6300	0.08/0.33/0.03	0.22
11	2.14	2.14	14.4	1.0	0.500	0.9	17.3	4.2	0.30	13.10	0.27/0.66/0.06	5	12.51	15600	6400	0.14/0.44/0.08	0.09
12	1.94	1.94	12.9	1.0	0.500	0.9	13.9	3.8	0.46	11.93	0.23/0.51/0.26	31	10.05	14800	6100	0.12/0.30/0.28	0.06
13	1.94	1.94	16.0	1.2	0.500	0.9	21.3	3.8	0.63	12.98	0.23/0.53/0.24	20	10.37	12800	5800	0.07/0.22/0.16	0.16

$M_{\rm targ}$ and $M_{\rm proj}$: mass of target and projectile; V_i: impact velocity in km/s and as factor of mutual escape velocity; b: impact parameter; x_0: initial separation in terms of sum of radii; Q_S: geometrically-adjusted specific impact energy (see SOM of[8]); $M_{\rm bnd}$: final bound mass; $M_{\rm tr}$: mass in disk; $R_{\rm tr}$: cylindrical radius of transition to disk; Mass frac.: bound mass fraction of core/mantle/water; $P_{\rm mw}$: pressure at mantle-water boundary, defined by equatorial level where water content exceeds 10wt%; $R_{\rm mw}$: equatorial radius of mantle-water boundary; $T_{\rm m}$, $T_{\rm w}$: temperature of mantle and water at mantle-water boundary; dIE/dKE$_0$: increase in internal energy of each layer in terms of initial kinetic energy of the event; $M_{\rm mix,m}$: mass fraction of silicate mantle mixed into water layer ($P < P_{mw}$).

Movie S1. Kovacevic_et_al_System3_Mixing(8000K).mov. Trajectory of system 3 with water (ice X) and rock (post-perovskite) showing rock and water mixing over a 3.72 ps DFT-MD simulation.

Movie S2. Kovacevic_et_al_ImpactSimulation_No1.mov. Giant impact trajectory showing equatorial slices of the material distribution during a graze-and-merge event. A 0.7 $M_\oplus$ body collides with a 4.7 $M_\oplus$ body at 23 km/s and a 30 degree angle. The colors represent the materials: iron (grays), rock (greens), and water (blues).

Movie S3. Kovacevic_et_al_ImpactSimulation_No2.mov. Giant impact trajectory showing equatorial slices of the material distribution during a graze-and-merge event. A 2.23 $M_\oplus$ body collides with a 2.96 $M_\oplus$ body at 15.8 km/s and a 40.5 degree angle. The colors represent the materials: iron (grays), rock (greens), and water (blues).

Dataset S1. EOS_30GPa_MgSiO3-pv_H2O-iceVIII.txt

Dataset S2. EOS_60GPa_MgSiO3-pv_H2O-iceX.txt

Dataset S3. EOS_120GPa_MgSiO3-ppv_H2O-iceX.txt

References

1. Wilson, H. F., Wong, M. L. & Militzer, B. Superionic to Superionic Phase Change in Water: Consequences for the Interiors of Uranus and Neptune. *Phys. Rev. Lett.* **110**, 151102, DOI: 10.1103/PhysRevLett.110.151102 (2013).
2. Fei, Y. *et al.* Melting and density of MgSiO3 determined by shock compression of bridgmanite to 1254GPa. *Nat. Commun.* **12**, 876, DOI: 10.1038/s41467-021-21170-y (2021).
3. Belonoshko, A. *et al.* High-Pressure Melting of $MgSiO_3$. *Phys. Rev. Lett.* **94**, 195701, DOI: 10.1103/PhysRevLett.94.195701 (2005).
4. Cheng, B., Bethkenhagen, M., Pickard, C. J. & Hamel, S. Phase behaviours of superionic water at planetary conditions. *Nat. Phys.* **17**, 1228–1232, DOI: 10.1038/s41567-021-01334-9 (2021).
5. Thompson, S. L., Lauson, H. S., Melosh, H. J., Collins, G. S. & Stewart, S. T. M-ANEOS v1.0. https://doi.org/10.5281/zenodo.3525030, DOI: 10.5281/zenodo.3509365 (2019).
6. Stewart, S. *et al.* The shock physics of giant impacts: Key requirements for the equations of state. In *AIP Conference Proceedings*, vol. 2272, 080003, DOI: 10.1063/12.0000946 (2020). 1910.04687.
7. Stewart, S. T. ANEOS Code Modification: Thermal model adjustment parameter. https://doi.org/10.5281/zenodo.3525030 (2019).
8. Lock, S. J. & Stewart, S. T. The structure of terrestrial bodies: Impact heating, corotation limits, and synestias. *J. Geophys. Res. Planets* **122**, 950–982, DOI: 10.1002/2016JE005239 (2017).
